# The gap and two-level differentiation in kindergarten education funding groups in China

**DOI:** 10.3389/fpsyg.2022.1020323

**Published:** 2022-11-29

**Authors:** Jiantao Chen, Xiao Wang, Xiang Luo

**Affiliations:** ^1^School of Economics and Management, Southwest Petroleum University, Chengdu, China; ^2^School of Accounting, Southwestern University of Finance and Economics, Chengdu, China; ^3^School of Economics and Management, Southwest Jiaotong University, Chengdu, China

**Keywords:** kindergarten, education funding, Gini coefficient, polarization, gap

## Abstract

This paper examines the disparity in kindergarten education funding between different groups from 2012 to 2018 through the Gini coefficient and decomposition techniques. The results show that: the Gini coefficient of inter-provincial kindergarten education funding increased from 0.2320 in 2012 to 0.2329 in 2018 during the observation period; the Gini coefficients between urban and rural areas and between regions are smaller than those between provinces, but it is noteworthy that the Gini coefficient between urban and rural areas is growing faster; the gap at this stage mainly comes from the internal gap between the two items of state financial education funding and career income. The internal disparity between urban and rural areas and between regions also contributes significantly to the overall disparity; there is no obvious polarisation in kindergarten education funding. Combined with the analysis results, the following recommendations are made: increase the investment in education funds, gradually reduce the proportion of school fee income in career income, financial resources should be tilted toward kindergartens in regions with relatively slow economic development, and financial resources should be appropriately distributed between public and private kindergartens.

## Introduction

China regards the development of education as a basic national policy, vigorously implements the strategy of rejuvenating the country through science and education and strengthening the country through talents, and strives to build a powerful country of human capital. From a social perspective, education can not only distribute economic welfare to the whole society but also narrow the socioeconomic gap and maintain social cohesion (Stucke, 2013). In the 14th Five Year Plan and the proposal for the long-term goal of 2035, the Communist Party of China points out that in the future, it is necessary to build a high-quality education system, promote educational equity, and the balanced development of compulsory education. Scholars mainly study educational equity by studying the allocation of human resources, material resources, and financial resources. Both human resources and material resources are based on financial resources (Ye and Zhou, 2017). Without the guarantee of education funds, there will be no high-quality human resources and material resources. As the material basis of school development, the balanced development of education funds can promote educational equity (Chang, 2015). Kindergarten education^1^ is the basis of China’s compulsory education and is highly profitable. Kindergarten education funds can enhance the accumulation of human capital and increase the contribution of human capital income (Cai et al., 2022). However, the Ministry of Education of China points out that kindergarten education is the weakest link in the process of the modernization of national education, and the problem of an unbalanced resource supply is quite obvious (Liu and Wu, 2021). If the gaps in education funding between different groups are too large, it will not be conducive to the balanced development of kindergarten education, and it will affect the quality of talent training among groups and harm China’s construction of a powerful human capital country. Therefore, it is of practical significance to analyze the education funding gap in China’s kindergartens.

Through literature review, it is found that since 2010, the fair distribution of kindergarten education funds has been widely concerned by scholars. Although government investment is the main source of kindergarten education funds (Jane and Anne, 2018), it is relatively limited and cannot support the vigorous development of kindergarten education (Wang, 2013). Moreover, it is allocated disproportionately between groups. This disproportion does not only exist among groups (inter-provincial, urban and rural, and regional) but also within groups (Song, 2011; Li et al., 2015; Wu, 2015; Zhao and Zhao, 2018; Lai and Chen, 2021). Scholars have found that the level of economic development is the main reason for the disproportion between regions. Areas with strong financial resources have relatively adequate investments (Liu and Wu, 2021). In addition, the different emphases of government investment in education are also a reason for the gap. The government investment in education is tilted toward urban public kindergartens, while private kindergartens have relatively insufficient investments (Liu, 2019).

From the perspective of research methods, scholars used various methods such as the Theil coefficient, Gini coefficient, and efficiency coefficient to study the gap in education funds between different groups in China’s compulsory education before 2016, and found through econometric models that the size of students, industrial structure, local financial capacity, economic development level, and other factors have a significant impact on the gap in education funds (Yao and Xu, 2016; Wang and Yan, 2017; Zhao and Liu, 2017; Zheng and Yue, 2017; Zhou et al., 2019; Geng, 2020). In addition, scholars also studied the improvement of educational quality (Yang and Li, 2015; Liu et al., 2021) and teacher training (Zhang and Guan, 2019; Liu and Zheng, 2021) in kindergartens; Some scholars also discussed the kindergarten rating system (Wu, 2021), access to education (Huang and Chen, 2014), equalization of education services (Zhuang and Zhu, 2018) and other issues from the perspective of equity. Scholars pointed out that the biggest influencing factor in these problems is how to rationally allocate education funds among regions.

To sum up, most of the current studies focus on the gaps in education funds from government investment, lacking comprehensive discussion of other sources, and the data are mostly from the years before 2016. It is also found that although the Gini coefficient has already been used in previous studies, it is insufficient to describe the gap in education funds only by using the Gini coefficient. Due to the complexity of the sources of education funds, it is also necessary to find the internal factors that affect the gap in education funds. Only by finding the internal factors, can we fundamentally overcome the weaknesses, effectively lessen the adverse effects caused by the widening gap, and optimize the distribution system of kindergarten education funds.

Because of this, this paper uses the Gini coefficient and decomposition technology to study the investment in China’s kindergarten education from 2012 to 2018, focusing on the following issues: (1) what is the current situation and trend of the gap in China’s kindergarten education investment? (2) what are the key factors that affect the gaps between investments in provincial, regional, urban and rural kindergartens? (3) is there a polarization in kindergarten education funding? and (4) how to optimize the current distribution system of kindergarten education funds?

The main contributions of this paper are as follows: First, it expands the research scope of the gap in education funding. Most of the existing literature focuses on government investment in education funds. This paper analyzes the gap between groups with different sources of education funds. Second, it expands the research methods of the gap in educational funds investment. Most of the existing studies apply the Thiel coefficient, efficacy coefficient, and econometric model. Although the Gini coefficient is also used, the gap is measured only by calculating the Gini coefficient. This paper comprehensively uses a variety of Gini coefficient decomposition techniques, calculates the polarization index to study the gap in kindergarten education funding investment, which can clarify the impact of different internal factors on the overall gap, and reveals the internal factors affecting the gap in education funding. It is expected to provide a methodological reference for the research on the gap in educational investment in other stages. Third, it provides some inspiration for improving the distribution system of educational funds in the stage of kindergarten and promoting the fairness of the allocation of financial resources in kindergarten education. This paper determines the extent to which different internal factors exert their impact on the gap in education funding, and puts forward some pertinent suggestions based on the research results, which can help the government create more effective policies.

## Sources of education funding data and research methods

### Selection of data

The basic data of this paper are from the *China Educational Finance Statistical Yearbook and the Educational Statistics Yearbook of China*. Since the content of the data of “general public budget education funds” has been changed from 2012, this paper selects the annual data of kindergarten education funds in 31 provinces, autonomous regions, and municipalities from 2012 to 2018 in *the China Educational Finance Statistical Yearbook*. According to sources, the total amount of education funds in different provinces and autonomous regions can be divided into national financial education funds, input from the founders of private schools, donations, and other education funds. The national financial education funds are composed of three parts: education funds arranged in the general public finance budget, education funds arranged in the government fund budget, and appropriations from state-owned and state-owned holding enterprises founding schools. Within the province, there are urban kindergartens and rural kindergartens. The number of students in different types of kindergartens comes from the *Educational Statistics Yearbook of China.* The education expenditure per student is the sum of the education expenditure of different types of kindergartens divided by the number of students in kindergartens.

### Methods of measuring gaps

In this paper, the Gini coefficient is used to measure the gap between different groups in kindergarten education funding, mainly because the Gini coefficient is widely used to measure the degree of inequality between groups. Moreover, with the advancement of the decomposition technology of the Gini coefficient, this paper can also use it to analyze the main factors affecting the gap in education funding between groups. The specific calculation method is as follows:

#### Calculation methods for different types of Gini coefficients

The formula for calculating the Gini coefficient for education funding between different groups is as follows:


(1) $$G_{edu}=\frac{1}{2n^{2}\mu }\overset{N}{\underset{i=1}{\sum }}\overset{N}{\underset{j=1}{\sum }}\left|a{\omega_{edu}}_{,i}-a{\omega_{edu}}_{,j}\right|$$

In the formula, represents sentsthe Gini coefficient of the average education funding between groups, n represents the number of students in each group, N represents the number of groups, μ represents the overall capital education funding and $a{\omega_{edu}}_{,i}$ and $a{\omega_{edu}}_{,j}$ represent the average education funding of the ith and jth groups, respectively. This formula is used in the calculation of the Gini coefficient between provinces, within regions, between regions, and between urban and rural areas.

#### The contribution of internal gaps from different sources to the gap between groups and the incremental decomposition of gaps between groups

To better analyze the impact of different sources of education funding on the income gap between groups, the Gini coefficient of inter-group education funding can be decomposed according to the structure of income sources using the following formula:


(2) $$G_{edu}=\overset{X}{\underset{m=1}{\sum }}\theta_{m}G_{m}^{\prime }=\overset{X}{\underset{m=1}{\sum }}\theta_{m}R_{m}G_{m}$$

$G_{edu}$ represents the Gini coefficient for inter-group education funding, $G_{m}^{\prime }$ represents the concentration index of education funding for the mth source, i.e., the pseudo-Gini coefficient; $\theta_{m}$ indicates the proportion of education funding from the mth source in total education funding, and X indicates the number of specific sources of income from a certain education funding. This formula is used to decompose the total income source of education funds and the source of financial education funding income.

$R_{m}$ refers to the Gini Correlation Coefficient proposed by Lerman and Yitzhaki (1985). Taking interprovincial as an example, this paper studies 31 provincial units, so q = (1, 2,…,31), then the education funding of the mth source of the qth provincial unit is $y_{qm}$.


(3) $$R_{m}=\frac{\sum_{q=1}^{31}\left(q-\frac{31+1}{2}\right)y_{qm}}{\sum_{q^{\prime }=1}^{31}\left(q^{\prime }-\frac{31+1}{2}\right)y_{q^{\prime }m}}=\frac{cov\left(y_{m},q\right)}{cov\left(y_{m},q'\right)}$$

Then, the contribution of the internal gap in education funding from the mth source to the gap in education funding between groups can be expressed as $\frac{\;\theta_{m}G_{m}^{\prime }}{G_{edu}}$.

#### Decomposition of the increment of the Gini coefficient between groups by the average education expenditure of students, the ranking of groups, and the student share

This analysis method was proposed by Chotikapanish and Griffiths, and improved by Hong, and this paper uses the improved method to decompose the increment of the Gini coefficient of inter-group education funding in 2012–2018 according to the education funding, group ranking, and student share of each group (Chotikapanish and Griffiths, 2001; Hong and Li, 2006). The formula is as follows:


(4) ΔG=ΔW+ΔQ+ΔP

In this formula, ΔW represents the change in the Gini coefficient caused by the change in education funding of each group under the fixed base period level of the group ranking and the student share; ΔQ indicates the change in the Gini coefficient due to changes in the ranking of groups due to the increase in education funding for each group; ΔP represents the change in the Gini coefficient caused by the change in student share caused by the change in education funding and inter-group ranking of each group fixed at the base period level.

#### Decomposition of inter-group Gini coefficients by different regions

Regarding the Gini coefficient group decomposition method, Scholars such as Mookherjee and Shorrocks (1982), Shorrocks (1984), and Yao (1997) have conducted in-depth explorations, and this paper will carry out multigroup decomposition under eight comprehensive economic zones.^2^ The decomposition formula is as follows:


(5) $$G_{edu}=G_{g}+\overset{M}{\underset{j=1}{\sum }}\theta_{j}P_{j}G_{j}+G\left(f\right)$$

In this formula, $G_{g}$ represents the Gini coefficient between regions, $\theta_{j}$ is the proportion of education funding in the group j to the total education expenditure, $P_{j}$ is the proportion of the number of students in the group j to the total student data, $G_{j}$ represents the Gini coefficient within the j group, and G(f) is the cross term. The meaning of the cross item refers to the degree of overlap in the distribution of education funding income between each group, and if there is no overlap at all, the cross item is 0.

#### A method of measuring the polarization of education funding

Aiming at the two-level differentiation of education revenue, this paper uses the following formula to measure (Hong and Li, 2007):


(6) $$W=\frac{2a\left(G_{B}-G_{W}\right)}{b}$$

In the formula, a and b are, respectively, the arithmetic average and median of education funding in 31 provinces, autonomous regions, and municipalities directly under the Central Government; $G_{W}$ is the weighted average of the Gini coefficient within the province, reflecting the contribution of the gap in the province to the overall gap; and $G_{B}$ is the Gini coefficient calculated by the average expenditure of each province and it reflects the contribution of the inter-provincial gap to the overall gap.

## The overall situation of kindergarten education funds in China

Table 1 reflects the average education funding for kindergarten students in China. It can be seen that since the implementation of the Kindergarten Education Action Plan in 2011, with the strong support of the central and local governments at all levels, the average education funding of kindergarten students in China has increased significantly during the study period. The average education expenditure of students increased from 4080.37 yuan in 2012 to 7886.77 yuan in 2018. It can be seen from the growth rate of the total investment that the total investment in 2012 increased by 37.18% compared with 2011, which is related to the target of 4% of the gross domestic product in 2012 in the “Twelfth Five-Year Plan” formulated by China in 2011. The growth rate from 2013 to 2019 remained around 11.61%.

**Table 1:** Average education expenditure and its growth of kindergarten students.

	2012	2013	2014	2015	2016	2017	2018
Education per student expenditure	4080.37	4513.98	5057.77	5690.13	6351.66	7078.16	7886.77
Growth rate	37.18%	10.63%	12.05%	12.50%	11.63%	11.44%	11.42%

China Educational Finance Statistical Yearbook and Educational Statistics Yearbook of China, the same below.

Table 2 reflects the proportion of income from different sources of kindergartens in their education funding. On average, about 47.81% of the education funding of kindergartens in China comes from the state’s financial appropriations; 48.46% comes from undertaking revenue, and 95% of undertaking revenue is tuition fees, and this proportion exhibits an upward trend year by year. In other words, a large part of China’s kindergarten education revenue relies on tuition fees, which also explains why kindergarten tuition fees have generally been so high in recent years that in some areas they are even higher than university tuition fees.

**Table 2:** Proportion of different sources of kindergarten education funding.

	State financial expenditure on education	Undertaking revenue	The investment made in private schools by their founders	Other education funding	Donation income
2012	49.71%	45.54%	2.68%	1.78%	0.28%
2013	49.05%	46.66%	2.28%	1.84%	0.17%
2014	45.59%	50.65%	2.03%	1.62%	0.12%
2015	46.68%	49.86%	1.95%	1.39%	0.12%
2016	47.30%	49.38%	1.81%	1.39%	0.11%
2017	48.02%	48.74%	1.76%	1.38%	0.09%
2018	48.30%	48.41%	1.83%	1.38%	0.07%

## Gini coefficient and decomposition of intergroup kindergarten education funding

### Gini coefficient and change trend of kindergarten education funding between groups

To reflect the gap between different groups of kindergarten education funds, this paper calculates the Gini coefficient of kindergarten education funds between different groups from 2012 to 2018 according to formula (1), and the results are shown in Table 3. The Gini coefficient for interprovincial kindergarten education funding fluctuated little during the study period, but there was a slow upward trend from 2016 to 2018—it rose from 0.2298 in 2016 to 0.2329 in 2018. The Gini coefficient for urban and rural kindergarten education funding increased by 98.39% during the study period. Only 0.0994 in 2012, it increased year by year, reaching a peak of 0.2032 in 2017, and then dropped to 0.1972 in 2018. The Gini coefficient for interregional funding for kindergarten education declined in fluctuations, with a maximum of 0.2486 in 2014 and a minimum of 0.1596 in 2016.

**Table 3:** Gini coefficient for intergroup funding for kindergarten education.

	2012	2013	2014	2015	2016	2017	2018
Inter-provincial	0.2320	0.2438	0.2486	0.2293	0.2298	0.2312	0.2329
Between urban and rural areas	0.0994	0.1025	0.1717	0.2018	0.2023	0.2032	0.1972
Inter-regional	0.1672	0.1818	0.1827	0.1629	0.1596	0.1637	0.1619

From the Gini coefficient of education funding between different groups, the gap between provinces is the most significant. Therefore, the next part of this paper will explore the influencing factors that cause the gap in inter-provincial education funding income, starting from the different sources of education funding.

### Gini coefficients of different sources of kindergarten education funding and their contribution to the overall gap

#### Gini coefficient for different sources of kindergarten education funding

According to formula (1), the internal Gini coefficient of different sources of inter-provincial kindergarten education funding can be calculated, and the results are shown in Table 4. Overall, among the sources of income of kindergartens, the largest inter-provincial gap lies in donation income. The average Gini coefficient was 0.5752; the maximum value was 0.6345 in 2015, and then it declined year by year. The main reason for the excessive value of the project is that the *per capita* value of the project in Beijing, Guangdong, and Chongqing is much higher than that of other provinces. The project with the smallest inter-provincial gap is career income—the maximum value does not exceed 0.2500, which is the smallest gap among the five projects. The gap between other education funding projects is also relatively large, except for in 2012; the rest of the years saw a Gini coefficient higher than 0.5000. The gap between the national financial education funding and the investment of the founders in private schools is relatively small, but overall, it shows an upward trend in fluctuations.

**Table 4:** Gini coefficients for different sources of interprovincial education funding.

	2012	2013	2014	2015	2016	2017	2018
State financial expenditure on education	0.3332	0.3493	0.374	0.3497	0.3645	0.3768	0.3731
The investment made in private schools by their founders	0.3426	0.3266	0.3647	0.3611	0.3518	0.3802	0.3663
Donation income	0.5663	0.5623	0.5801	0.6345	0.6149	0.5447	0.5236
Undertaking revenue	0.2492	0.2458	0.2266	0.2161	0.2184	0.2304	0.2283
Other education funding	0.4749	0.5584	0.5703	0.5107	0.5146	0.5148	0.5247

#### The contribution to the overall gap made by kindergarten education funding of different sources

To study the extent to which different sources of education funding affect the overall gap, this paper calculates the contribution of the internal gap of different sources of education funding to the overall gap according to formula (2), and the results are shown in Table 5.

**Table 5:** Extent to which internal gap in education funding of different sources contributes to the overall gap.

	2012	2013	2014	2015	2016	2017	2018
State financial expenditure on education	58.68	58.75	59.52	61.66	64.89	67.42	65.46
Investments made in private schools by their founders	0.62	0.9	1.65	1.37	1.15	1.46	1.72
Donation income	0.52	0.29	0.16	0.16	0.2	0.15	0.1
Undertaking revenue	37.7	37.11	35.46	34.04	31.03	28.21	30.02
Other education funding	2.47	2.95	3.21	2.77	2.73	2.76	2.7

On the whole, the contribution rate of the internal gap in the national financial education funding project to the overall gap has increased year by year, from 58.68% in 2012 to 65.46% in 2018. The contribution rate of undertaking revenue showed a slow downward trend, with the contribution rate in 2012 being 37.70% and falling to 30.02% in 2018; None of the remaining three projects contributed more than 5 percent. Therefore, at the present stage, the gap in kindergarten education funds mainly comes from the internal gap between national financial education funds and undertaking revenue, especially the former, which not only have the highest contribution rate but also rise year by year. Therefore, this paper will further decompose the national financial education funding project.

### Gini coefficients of financial education funding for kindergartens from different sources and their contributions to the overall gap

#### Gini coefficient of different sources of financial education funding for kindergartens

In this paper, the internal gaps in the financial education funding projects of different sources are calculated according to formula (1), and the results are shown in Table 6. The Gini coefficient of inter-provincial national financial education funding projects from all sources showed a certain degree of growth between 2012 and 2018. Among them, the Gini coefficient of education funding arranged in the general public finance budget is the smallest, with an average value of 0.3549; the Gini coefficient of education funding items arranged by the government fund budget has seen the largest increase, from 0.5442 in 2012 to 0.6579 in 2018; the Gini coefficient of enterprise appropriations in state-owned and state-controlled enterprises running schools is the largest, and it has reached 0.7079 by 2018.

**Table 6:** Gini coefficients for different sources of interprovincial financial education funding.

	2012	2013	2014	2015	2016	2017	2018
Education funds budgeted by the general public budget	0.3179	0.3244	0.3714	0.3511	0.3660	0.3787	0.3748
Education funds budgeted by Government funds	0.5442	0.5729	0.6015	0.6334	0.5724	0.6138	0.6579
Appropriation by State-owned and its holding enterprises when running schools	0.6237	0.6346	0.6725	0.6482	0.6765	0.6790	0.7079

#### The contribution to the overall gap made by different sources of financial education funding for kindergartens

Using formula (2), it is possible to calculate the extent to which the internal gap between different sources of financial education funding contributes to the overall gap in financial education funding, and the results are shown in Table 7. The contribution rate of the internal gap in education funding in the general public budget to the overall gap has increased year by year, from 81.07% in 2012 to 98.8% in 2018. The contribution rate of the remaining three projects has shown a downward trend year by year, and in recent years, it has not exceeded 1%. From the perspective of rural and urban areas, the contribution rate of education funding projects in the general public budget has also increased to 99.06 and 98.54%, respectively, in 2018. It can be seen that at the present stage, the gap in financial education funding for kindergartens is almost entirely due to the internal gap in education funding arranged in the general public budget.

**Table 7:** Extent to which internal gaps in financial education funding contribute to the overall gaps.

	2012	2013	2014	2015	2016	2017	2018
The general public budget allocates funds for education	81.07	77.35	90.41	99.09	99.33	99.32	98.80
Government fund budget arrangements for education	6.63	9.44	8.39	0.25	0.22	0.38	1.12
State-owned and its holding enterprises in the running of enterprises to allocate funds	1.33	1.22	1.20	0.66	0.45	0.29	0.09

### Incremental decomposition of gaps

#### Decomposition of the overall gap in education funding

According to formula (4), this paper breaks down the total income of education funding and the increase of its largest source of financial education funding from 2012 to 2018 (see Table 8). From 2012 to 2018, the Gini coefficient of kindergarten education funding increased from 0.2320 to 0.2329, and the increment was decomposed according to the change in *per capita* input of education funding in each province (ΔW), the change in the inter-provincial ranking (ΔQ), and the change in student share (ΔP), and the results were: ΔG/G was 0.38%, ΔW/G was −3.56%, ΔQ/G was 4.42%, and ΔP/G was −0.43%. That is to say, the Gini coefficient increased by 0.0009 because the change in the average student input of education funds in each province caused it to decrease by 3.56%, the change in student share caused it to decrease by 0.43%, but the change in inter-provincial ranking caused it to increase by 4.42%. Similarly, what caused the Gini coefficient of national financial education funding for an average kindergartener to increase by 10.72% in 2018 compared with the number in 2012 was the positive impact of changes in inter-provincial ranking, student share, and national financial education funding per student, which has reduced it by 1.07%. Therefore, we should increase financial support for backward provinces and increase the amount of investment in education per student in backward provinces, to reduce the gap between provinces.

**Table 8:** Gap incremental decomposition results.

	ΔG/G	ΔW/G	ΔQ/G	ΔP/G
Education funding	0.38%	−3.56%	4.42%	−0.43%
Financial education funding	10.72%	−1.07%	9.30%	2.47%

### Gini coefficient and decomposition of kindergarten education funds in different regions

#### Gini coefficient of interregional education funding and its contribution to overall disparities

Table 9 shows the Gini coefficients for kindergarten education funding in different regions and the contribution of intra-regional disparities to the overall disparities, calculated based on formulas (1), (5). It can be seen from the table that from 2012 to 2018, the Gini coefficient between different regions decreased in fluctuations, with a maximum of 0.1827 in 2014 and a minimum of 0.1596 in 2016; according to the data in the table, the Gini coefficient in the northern coast was the largest, with an annual average of 0.2900 during the study period, and the Gini coefficient in the southern coast, northeast China and the middle reaches of the Yangtze River was small, with annual averages of 0.0282, 0.0445, and 0.0612, respectively. From the perspective of the trend of change, only the Gini coefficient in the middle reaches of the Yellow River showed a fluctuating decline, but the decline was only 1.09%; The Gini coefficient in other regions showed a fluctuating upward trend, such as the average difference between the students in the southwest region which rose from 4898.95 yuan in 2012 to 7678.84 yuan in 2018. From the perspective of contribution rate, the contribution rate between regions is the largest, with an annual average of 71.56%, and the minimum annual average contribution rate within the group is 0.04% in the northeast region, and the maximum value is only 2.57% (northern coastal area).

**Table 9:** Gini coefficients for intra-regional education funding and their contribution to overall disparities.

	2012	2013	2014	2015	2016	2017	2018
Northeast	0.0486	0.0461	0.0292	0.0326	0.045	0.0542	0.0558
	0.06%	0.05%	0.03%	0.03%	0.04%	0.04%	0.04%
Eastern coastal	0.1466	0.1503	0.1575	0.1581	0.1793	0.1815	0.1801
	1.60%	1.53%	1.51%	1.55%	1.68%	1.65%	1.62%
Southern coastal	0.026	0.0263	0.0269	0.0327	0.0224	0.0338	0.0293
	0.22%	0.22%	0.24%	0.31%	0.22%	0.32%	0.28%
Northern coastal	0.271	0.3097	0.3052	0.2739	0.2794	0.294	0.297
	2.64%	2.96%	2.60%	2.45%	2.36%	2.38%	2.60%
The middle reaches of the Yellow River	0.2207	0.1837	0.1889	0.1914	0.2001	0.1664	0.167
	1.92%	1.55%	1.61%	1.75%	1.76%	1.39%	1.36%
The middle reaches of the Yangtze River	0.0563	0.0509	0.0629	0.0825	0.0595	0.0561	0.0605
	0.47%	0.42%	0.49%	0.72%	0.53%	0.47%	0.50%
Southwest	0.081	0.0995	0.0914	0.0833	0.0933	0.1036	0.1053
	0.89%	0.96%	0.91%	0.98%	1.11%	1.24%	1.25%
Northwest Territories	0.0836	0.1134	0.1542	0.1243	0.14	0.1598	0.1174
	0.07%	0.10%	0.14%	0.14%	0.22%	0.36%	0.24%
Inter-regional	0.1672	0.1818	0.1827	0.1629	0.1596	0.1637	0.1619
	72.07%	74.57%	73.49%	71.04%	69.45%	70.80%	69.51%
The remaining items	0.0465	0.043	0.0472	0.0482	0.052	0.0493	0.0526
	20.06%	17.65%	18.99%	21.03%	22.64%	21.34%	22.59%

#### The gap in education funding between urban and rural areas and its contribution to the overall gap

Table 10 shows the Gini coefficient of education funding for an average rural or urban kindergarten student calculated based on formulas (1) and (5) and the contribution of their respective internal gap to the overall gap. In the area divided into villages and towns, the Gini coefficient of rural internal education funding is decreasing, with an annual average of only 0.0347; its contribution rate to the overall gap is on average 14.72%, showing a gradually decreasing trend. Specifically, the average education funding of students in Inner Mongolia (the education funding per student rose from 9619.25 yuan in 2012 to 45249.42 yuan in 2018) and Jilin (from 8019.77 in 2012 to 32083.48 yuan in 2018) and other provinces are growing, while the average education funding of Shanghai students, which widened in 2012, remained at 140,000 yuan during the study period. The gap within cities and towns shows the opposite trend as the internal gap in the average education funding of urban students gradually increased from 0.1043 in 2012 to 0.1377 in 2018, and the contribution rate also shows a year-on-year growth trend. Specifically, the regions and provinces that originally had small interprovincial gaps in the average education funding for urban students in 2012 have gradually widened their gap in recent years. For example, the education funding per student in Beijing rose from 15680.98 yuan in 2012 to 33807.59 yuan in 2018 and that of Shanghai rose from 11,911.22 yuan in 2012 to 28,069.41 yuan in 2018, whereas the average education expenditure of students in Heilongjiang, Anhui, Hunan, Guangxi, Gansu, Ningxia, and other places has hardly increased, and some regions even exhibited a negative growth trend. In 2012, the average gap between rural and urban students in education was 8320.42 yuan, and by 2018, the gap increased to 18037.90 yuan, which led to the largest contribution rate of the average Gini coefficient between rural and urban students to the overall gap, with an annual average of 71.80%. In addition, it can be seen that the negative contribution of the remaining items increased significantly after 2014, which shows that a series of policies implemented by the state to reduce the regional and urban–rural gaps in education investment had played a positive role.

**Table 10:** Decomposition of the overall gap in different areas of villages and towns.

	2012	2013	2014	2015	2016	2017	2018
Rural *per capita* Gini coefficient	0.0376	0.0374	0.0374	0.0354	0.0338	0.0313	0.0297
	16.23%	15.34%	15.03%	15.45%	14.72%	13.52%	12.76%
Urban *per capita* Gini coefficient	0.1043	0.1105		0.1251	0.1269	0.1318	0.1377
	44.96%	45.33%	51.42%	54.57%	55.23%	56.99%	59.14%
The average Gini coefficient between urban and rural areas	0.0994	0.1025	0.1717	0.2018	0.2023	0.2032	0.1972
	42.84%	42.05%	69.06%	88.03%	88.04%	87.90%	84.66%
The remaining items	−0.0093	−0.0066	−0.0883	−0.1331	−0.1332	−0.135	−0.1317
	−4.03%	−2.71%	−35.51%	−58.04%	−57.98%	−58.41%	−56.57%

### Polarization

**Figure 1:**
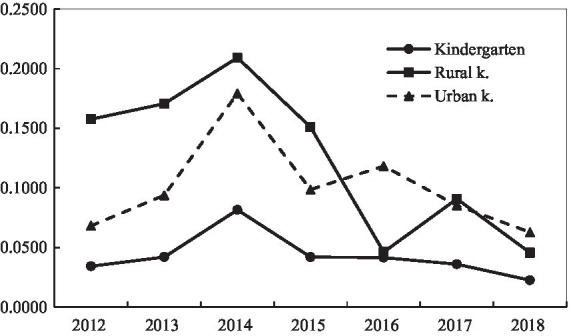
Polarization trend of total income for education.

Figure 1 is a polarization trend chart of the total income of kindergarten education funds calculated according to formula (6). From the figure, it can be seen that the polarization index of kindergarten education funds in 2012–2018 was not very large, the overall value was not large either and showed a downward trend; the minimum value was 0.0225 in 2018, and the maximum value was only 0.0815 (2014), which shows that there was no obvious polarization problem in kindergarten education funding. Rural kindergartens and urban kindergartens also exhibited the same trend; although the development trend of rural kindergartens and urban kindergartens was more consistent, the internal reasons for the development trend of the two were different. The average annual decline rate of the gap between the high-income education funding group and the low-income education funding group in rural kindergartens was greater than that of the inter-group gap of 1.03%, and the average annual decline in $G_{B}$was faster than $G_{w}$ leading to the overall downward trend. The average annual growth rate of urban kindergarten $G_{B}$ was 5.06%, while the average annual growth rate of $G_{w}$ was 5.99%, making the $G_{B}$ growth not as fast as $G_{w}$; but the difference between $G_{w}$ and$G_{B}$ was decreasing, which seemed to have led to an overall decline in polarization.

## Conclusion and policy recommendations

### Conclusion

During the observation period, the gap between kindergarten education funds between different groups (inter-provincial, urban–rural, and inter-regional) was widening, the largest gap was between provinces, and the fastest growth rate was between urban and rural areas, an increase of 98.39% in 2018 compared with 2012.

The gap between provinces in kindergarten education funds mainly comes from the internal gap between the two projects of national financial education funding and undertaking revenue, especially the national financial education funding project, which not only has the highest contribution rate, but also increases year by year, and the contribution rate has increased from 58.68% in 2012 to 65.46% in 2018. Almost all of the internal gaps in the state’s financial education funding projects stem from the internal gaps in the general public budget for education funding. The contribution rate of the internal gap in education funding in the general public budget to the internal gap in the national financial education funding project has increased year by year, from 81.07% in 2012 to 98.8% in 2018.

According to the results of decomposing the increment of interprovincial education funding and interprovincial financial education funding in 2012–2018, it is found that the change in the education funding per student will lead to a decrease in the Gini coefficient, the change in interprovincial ranking will lead to an increase in the Gini coefficient, and the effect of the change in student share is uncertain. Therefore, we should increase financial support for backward provinces and increase the *per capita* investment in education funds in backward provinces, to reduce the gap between provinces.

From the perspective of different regions, the internal gap in kindergarten education funding in the northern coastal area is the largest, with the Gini coefficient averaging 0.2900, but the average annual contribution rate of the internal gap to the overall gap is only 2.57%, and the interregional gap has the largest contribution rate to the overall gap, with an annual contribution rate of 71.56%.

From the perspective of urban and rural areas, the gap in kindergarten education funding within rural areas is decreasing; The gap within the town is gradually increasing, and the contribution rate of the gap within the town to the overall gap is also increasing year by year. The gap between urban and rural areas contributed the most to the overall gap, with an annual average of 71.80%.

Different from the research of Cai et al., based on the calculated polarization index, this paper finds that there is no obvious polarization problem in China’s kindergarten education funds as a whole.

### Policy recommendations

Increase the investment in education funds, and at the same time coordinate and adjust the inter-provincial gap in general financial education funds, and the specific use of funds should be allocated and used under the education funding plan for students, to avoid the unfair distribution of education funds and resources caused by the excessive income gap between the average education funds of each province.

Pay attention to the inter-provincial gap in the source of education funding income, and gradually reduce the proportion of tuition income in the total source of education funding in the undertaking revenue. Now there is a widespread phenomenon of excessive kindergarten tuition fees in various regions. According to the National Bureau of Statistics, the disposable income *per capita* in China was CNY35,000 in 2021. But in 2006, some kindergartens in first-tier cities such as Beijing and Shanghai charged more than CNY100,000 a year, and kindergartens charging over CNY5000 a month had also become common. According to the survey, more than 40% of rural parents think that kindergarten fees are too high. That the pressure of the admission threshold shifted to the parents of young children may implement the national three-child policy “difficult to implement,” and it may be difficult to attain the goal of “increasing the gross enrollment rate of preschool education to more than 90% in the 14th Five-Year Plan and the “Long-Range Objectives Through the Year 2035″ without solving the problem of high tuition fees.

Pay attention to the internal and inter-regional gaps in different groups. The level of regional economic development directly determines the total amount of educational resources and also directly affects the number of income sources of each part. Financial resources should be tilted to kindergartens in areas where economic development is relatively slow, such as the Northwest and the Northeast, especially to rural kindergartens with large internal gaps at the present stage. This will also help to alleviate the “U-” shaped feature of the inter-provincial gap in teachers’ salary cost per student (Tian and Du, 2021). Pay attention to the internal gap in urban kindergartens as well. The share of urban middle school students is decreasing, but the average education expenditure per student is gradually showing an uneven trend. Accelerating the development of inclusive private kindergartens requires appropriate allocation of financial funds in public and private kindergartens, which can help them withstand the test of force majeure on preschool education (Liu et al., 2021), reduce the emergence of whopping-price kindergartens, and alleviate the intra- and inter-group growth of high-income groups and low-income groups in urban kindergartens.

## Data availability statement

Publicly available datasets were analyzed in this study. This data can be found at: China Educational Funding Statistical Yearbook China Education Statistical Yearbook.

## Author contributions

JC: conceptualization, methodology, software, investigation, formal analysis, and writing—original draft. XW: formal analysis, writing, data curation, and writing—original draft. XL: visualization and investigation. All authors contributed to the article and approved the submitted version.

## Conflict of interest

The authors declare that the research was conducted in the absence of any commercial or financial relationships that could be construed as a potential conflict of interest.

## Publisher’s note

All claims expressed in this article are solely those of the authors and do not necessarily represent those of their affiliated organizations, or those of the publisher, the editors and the reviewers. Any product that may be evaluated in this article, or claim that may be made by its manufacturer, is not guaranteed or endorsed by the publisher.

## Footnotes

^1^In 2016, the Ministry of Education of the People’s Republic of China published the Work Rules for Kindergartens, which stipulates that the school-age children in kindergartens are generally 3–6 years old, and the school system is 3 years. Kindergarten education is an important part of basic education and the basic stage of school education system. The main task of kindergarten education is to implement the national education policy, follow the principle of combining care with education, follow the characteristics and laws of children’s physical and mental development, implement all-round education in moral, intellectual, physical and esthetic aspects, and promote the harmonious development of children’s physical and mental health.

^2^With reference to the Strategy and Policy for Coordinated Regional Development issued by the Development Research Center of the State Council, this study divides 31 provinces, municipalities and autonomous regions into eight comprehensive economic zones in four major sectors, namely, the Northeast Comprehensive Economic Zone (Liaoning, Jilin, Heilongjiang), the Northern Coastal Comprehensive Economic Zone (Beijing, Tianjin, Hebei, Shandong), the Eastern Coastal Comprehensive Economic Zone (Guangdong, Fujian, Hainan) Southern Coastal Comprehensive Economic Zone (Shanghai, Jiangsu, Zhejiang), Middle Yellow River Comprehensive Economic Zone (Shaanxi, Gansu, Ningxia, Shanxi, Henan), Middle Yangtze River Comprehensive Economic Zone (Sichuan, Chongqing, Hubei, Hunan, Anhui, Jiangxi), Great Southwest Comprehensive Economic Zone (Guangxi, Guizhou, Yunnan, Xizang) The Greater Northwest Comprehensive Economic Zone (Xinjiang, Qinghai, Inner Mongolia) (hereinafter referred to as the Northeast, the northern coast, the eastern coast, the southern coast, the middle reaches of the Yellow River, the middle reaches of the Yangtze River, the southwest, and the northwest respectively).
